# Bioactive Molecules from Extreme Environments III

**DOI:** 10.3390/md24060212

**Published:** 2026-06-15

**Authors:** Daniela Giordano

**Affiliations:** 1 Institute of Biosciences and BioResources (IBBR), National Research Council (CNR), Via Pietro Castellino 111, 80131 Napoli, Italy; daniela.giordano@ibbr.cnr.it or daniela-giordano@cnr.it; 2 Department of Marine Biotechnology, Stazione Zoologica Anton Dohrn (SZN), Villa Comunale, 80121 Napoli, Italy

Marine organisms represent an extraordinary source of chemically diverse natural products that confer key adaptive advantages, such as antimicrobial defense and communication within complex ecological networks. The MarinLit database, a major reference for marine natural product research, currently reports 44710 compounds as of May 2026, underscoring the vastness of this molecular repertoire [1]. In 2022 alone, 1417 new marine-derived compounds with significant biological activities were described, further highlighting the immense and still-expanding potential of marine biodiversity [2]. These molecules have long been exploited across multiple sectors, spanning pharmaceutical, cosmeceutical, and biotechnological applications, and they continue to attract increasing commercial interest. Consistent with this trend, the global market for marine-derived drugs, valued at USD 2770 million in 2024, is projected to reach USD 4959 million by 2031, reflecting steady and sustained growth [3]. Nevertheless, despite this remarkable chemical richness and economic potential, only a limited number of marine-derived compounds have been approved to date [4], and a substantial proportion of marine ecosystems remains largely unexplored. Among these, extreme marine environments remain one of the most largely unexplored and promising areas of marine biodiversity.

Extreme environments are characterized by exposure to intense and often fluctuating stressors, such as extreme temperatures, high salinity, pressure, radiation, or low oxygen availability. In marine systems, habitats including hydrothermal vents, hypersaline basins, polar seas, and deep-sea regions represent biodiversity hotspots where life persists close to its physiological limits. These conditions impose strong selective pressures that drive the evolution of highly specialized organisms and unique metabolic strategies, making these ecosystems rich sources of novel bioactive compounds. Despite their potential, exploring extreme marine environments remains challenging due to the need for specialized technologies and access to remote locations. Although recent technological advances have improved accessibility and accelerated research, the biodiversity of these systems is still far from being fully characterized. As such, extreme marine environments continue to represent one of the most promising frontiers for discovering structurally and functionally novel bioactive molecules.

The Special Issue “Bioactive Molecules from Extreme Environments III”, the third edition of this series in Marine Drugs, builds upon and continues the scientific framework established in the previous editions. Since its launch in 2019 (https://www.mdpi.com/journal/marinedrugs/special_issues/Extreme_Environments, accessed on 9 June 2026), followed by the second edition in 2020 (https://www.mdpi.com/journal/marinedrugs/special_issues/Extreme_Environments_II, accessed on 9 June 2026), this Special Issue series has aimed to consolidate and highlight the growing interest in marine organisms from extreme ecosystems, bringing together, over the years, key advances in the discovery of bioactive molecules from these challenging habitats. This third edition further strengthens this continuity, reflecting the sustained expansion of research in this field and the increasing recognition of extreme marine environments as invaluable sources of structurally diverse and functionally unique biomolecules. From deep-sea habitats to hypersaline systems and polar regions, these environments represent important reservoirs of largely unexplored biodiversity, where organisms have evolved sophisticated molecular strategies to cope with physical and chemical constraints close to the limits of life. This Special Issue comprises eleven contributions, including eight original research articles and three review papers. The next section provides a concise overview of the research findings alongside a synthesis of the current literature, offering readers a guided perspective to identify contributions relevant to their specific fields of interest. The individual works are listed in the List of Contributions.

Some contributions focuses on deep-sea ecosystems, highlighting their role as reservoirs of structurally unique and pharmacologically relevant metabolites. Contribution 1 by Desai et al. investigates deep-sea marine metabolites as promising anti-tubercular agents through a computational-aided drug discovery (CADD)-driven screening approach targeting the F420-dependent oxidoreductase, demonstrating the potential of marine-derived compounds from the deep sea as novel therapeutic leads. In Contribution 2, Olsen et al. identify anthoteibinenes F–Q, a series of new sesquiterpenes isolated from the Irish deep-sea coral *Anthothela grandiflora*, further expanding the chemical diversity known from deep benthic organisms and highlighting their bioactive potential. In Contribution 3, Kim et al., focusing on microorganisms adapted to nutrient-limited and dynamic high-seas conditions, describe the deep-sea bacterium *Galbibacter orientalis* strain ROD011, which produces extracts capable of modulating LPS-driven pro-inflammatory signaling, emphasizing the pharmacological relevance of high-seas microbial metabolites.

Other contributions address hypersaline and chemically extreme environments, where microorganisms have evolved highly specialized biochemical adaptations. Sabkhas, hypersaline ecosystems located along the Kuwaiti coastal zone, represent particularly harsh habitats that host diverse halophilic microorganisms with significant yet still underexplored biotechnological potential, especially in relation to enzymes capable of functioning under high-salinity conditions.

Within this context, in Contribution 4, Alagarsamy et al. describe a cobalt-activated halotolerant alpha-amylase from *Priestia* sp. W243, characterized by high specific activity and remarkable efficiency under saline conditions. The unique properties of this enzyme distinguish it from its conventional salt- or calcium-dependent counterparts, highlighting its suitability for industrial applications such as high-salt food processing, baking, brewing, and environmental remediation.

In parallel, Contribution 5 by Atak et al. explores microbial diversity and cultivation strategies in hypersaline ecosystems, such as the Sečovlje salt pans (Slovenia), particularly promising sources of novel bioactive compounds, as their microorganisms have evolved adaptations to desiccation and high-light-intensity stress. The study is focused on microalgal and cyanobacterial species from the Sečovlje salt pans, establishing a valuable resource for future bioprospecting and sustainable biotechnological exploitation.

Other studies highlight Antarctic environments, where extreme cold and environmental variability drive unique metabolic and evolutionary adaptations. In Contribution 6, Nagoth et al. report the synthesis of bioactive nickel nanoparticles using bacterial strains from an Antarctic consortium, including *Marinomonas* sp. ef1, *Rhodococcus* sp. ef1, *Pseudomonas* sp. ef1, *Brevundimonas* sp. ef1, and *Bacillus* sp. ef1, highlighting the potential of cold-adapted microorganisms for innovative nanobiotechnological applications. These nanoparticles exhibit pronounced antimicrobial activity, suggesting promising applications in developing new strategies for controlling and managing infectious diseases. 

In Contribution 7, Trentin et al. investigate the bioprospecting potential of the Antarctic diatoms *Craspedostauros ineffabilis* and *Craspedostauros zucchelli*, highlighting their ability to produce bioactive compounds and underscoring the relevance of microalgae in polar ecosystems. Expanding this perspective, in Contribution 8, Pucciarelli et al. explore molecular evolution and adaptation strategies in the marine Antarctic ciliate *Euplotes focardii*, providing insights into cold-adapted enzyme functionality and drug binding mechanisms and bridging evolutionary biology with applied biotechnology.

Finally, a set of contributions emphasizes marine biotechnology and sustainable applications. In Contribution 9, Pereira and de Carvalho focus on improving bioprocess conditions for producing the bioactive pigment prodigiosin, with anticancer and antibacterial properties, using a marine strain of *Serratia rubidaea* isolated from a shallow-water hydrothermal vent, demonstrating how process optimization can enhance metabolite yield and industrial relevance.

In Contribution 10, Ruginescu and Purcarea review plastic-degrading enzymes from marine microorganisms, including those from extreme environments, such as hot springs, hydrothermal vents, deep-sea regions, and Antarctic seawater, highlighting their potential role in recycling technologies and their relevance for addressing global environmental challenges through enzyme-based solutions. 

Contribution 11 by Benhadda et al. compares marine and non-marine bacterial exopolysaccharides, including those from extreme environments, emphasizing their properties and potential use in skincare applications and cosmeceutical formulations.

Collectively, the studies included in this Special Issue demonstrate the remarkable diversity and versatility of marine-derived bioactive molecules and underline the importance of extreme environments as sources of innovation. They demonstrate how adaptations in harsh ecological conditions foster the emergence of unique biochemical functions with broad applicability across multiple sectors. At the same time, this collection of studies emphasizes the need for the continued exploration of these environments, the development of advanced methodologies for bioprospecting, and the integration of sustainable approaches to harness marine resources.

By bringing together contributions from different disciplines and ecological contexts, this Special Issue offers a comprehensive and updated perspective on the field, fostering new insights and future research directions in marine drug discovery and biotechnology.

As a Guest Editor of the third edition of this Special Issue, I warmly thank the authors for their high-quality contributions and the reviewers and Marine Drugs editorial team for their invaluable support. These studies demonstrate that extreme environments are not marginal frontiers but engines of molecular innovation. I hope this collection will serve as a definitive reference and a catalyst for interdisciplinary research that accelerates discovery, translation, and sustainable impact in marine biotechnology and drug development.
